# Thromboendarterectomy in unilateral chronic thromboembolism in order to improve hemodynamic and functional parameters

**DOI:** 10.1590/1806-9282.20250785

**Published:** 2026-10-12

**Authors:** Ilker Sengul, Demet Sengul

**Affiliations:** 1 Giresun University, Faculty of Medicine, Division of Endocrine Surgery – Giresun, Turkey.; 2 Giresun University, Faculty of Medicine, Department of General Surgery – Giresun, Turkey.; 3 Giresun University, Faculty of Medicine, Department of Pathology – Giresun, Turkey.

Dear Editor,

We have read the research article "Unilateral Chronic Thromboembolic Pulmonary Disease: Do Patients Benefit From Thromboendarterectomy with great interest." This utilitarian research seems to demand a determination that sheds light on the efficacy of the pulmonary artery bypass graft in order to treat this unusual pathologic phenomenon, unilateral chronic thromboembolism pulmonary disease (CTEPD), though certain restrictions are ineludible, which affects the soundness of the outcomes in the issue of thromboembolism^1-3^. Rashidi et al.^1^ reported this multicentre study, involving the cases with unilateral CTEPD from three referral centers, evaluating their demographic information, 6-min walk test distance (6MWD), New York Heart Association (NYHA) functional class, and hemodynamics. The authors emphasized a significant progression in the mean pulmonary artery pressure and pulmonary vascular resistance. They also remarked on a substantial promotion in the NYHA functional class and 6MWD. Rashidi et al.^1^ re-evaluated the cases for at least 3 months after discharge, and pre- and postoperative 6MWD data were performed. The authors analyzed the pathology in the studied population using the 6MWT 3 months after surgery, and the mortality was appraised in the hospital and 1 year after surgery. The study has several notable strengths. Firstly, it focuses on a very rare condition, unilateral CTEPD, thereby contributing valuable data to a scarcely populated area of research. The multicenter design enhances the generalizability of the findings. Furthermore, the inclusion of patients with CTEPD without pulmonary hypertension (PH), who showed symptomatic improvement despite normal resting hemodynamics, is a significant contribution. The authors also rightly emphasize the importance of differentiating unilateral pulmonary artery involvement from other etiologies.

However, the study also has some flaws and limitations that warrant consideration. Initially, the sample size, while significant for such a rare condition, remains relatively small, which may limit the number and affect the statistical power of subgroup analyses, such as the comparison between lobar and segmental involvement. Secondly, the lack of postoperative right heart catheterization data at 3 months for all the cases, as well as the use of different cardiac output measurement methods across the centers, could introduce variability in the assessment of haemodynamic outcomes. Furthermore, the study does not delve into the etiology of unilateral involvement. While the authors mention previous reports suggesting a link between inherited thrombophilia and unilateral CTEPH in young women, the current study only assessed for antiphospholipid syndrome^1^. Further investigation into other potential risk factors, such as other thrombophilias and the role of estrogen-based medications in women, would be beneficial. Nevertheless, would the utilization of long-term follow-up beyond a 1-year period for all the studied participants alter the outcome(s) of this study? Furthermore, would harnessing larger sampling prior to the procedure transmute the desinence(s) of this work?

In conclusion, this study provides substantial evidence supporting the safety and efficacy of pulmonary artery bypass graft in improving symptoms and haemodynamic parameters in carefully selected patients with unilateral CTEPD, including those without PH. However, the limitations of the study, particularly the small sample size, should be considered when interpreting the results^1^. Larger, prospective studies with standardized follow-up protocols are necessary to validate these findings further and to gain a better understanding of the pathophysiology and long-term outcomes of unilateral CTEPD. It is also crucial to continue to refine the diagnostic work-up to differentiate unilateral CTEPD from other conditions affecting the pulmonary arteries. This issue merits further investigation. We thank Rashidi et al.^1^ for their valuable study on thromboembolism^2,3^.

## Data Availability

The datasets generated and/or analyzed during the current study are available from the corresponding author upon reasonable request.
